# Black electrochromic ink with a straightforward method using copper oxide nanoparticle suspension

**DOI:** 10.1038/s41598-023-34839-9

**Published:** 2023-05-13

**Authors:** Chan Yang Jeong, Hiroshi Watanabe, Kazuki Tajima

**Affiliations:** National Institute of Advanced Industrial Science and Technology (AIST), 1-1-1 Higashi, Tsukuba, Ibaraki 305-8565 Japan

**Keywords:** Nanoparticles, Synthesis and processing

## Abstract

Electrochromic (EC) materials for smart windows must exhibit a dark colour and block visible light (wavelength = 380–780 nm) to reduce environmental impact. In particular, black tones are also desired, and there are many reports of attempts to create these dark tones using organic materials such as polymers. However, their fabrication methods are complicated, expensive, and may even use hazardous substances; moreover, they are often not sufficiently durable, such as upon exposure to ultraviolet light. There are some reported cases of black materials using the CuO system as an inorganic material, but the synthesis method was complicated and the functionality was not stable. We have found a method to synthesize CuO nanoparticles by simply heating basic copper carbonate and adjusting the pH with citric acid to easily obtain a suspension. The formation and functionality of CuO thin films were also demonstrated using the developed suspension. This research will enable the creation of EC smart windows using existing inorganic materials and methods, such as printing technology, and is the first step towards developing environment-friendly, cost-effective, and functional dark inorganic materials.

## Introduction

Electrochromic (EC) materials exhibit reversible optical properties via electrochemical redox reactions, making it possible to control the transmittance and absorption in the near-infrared (NIR) and visible regions^1,2^. Owing to this unique ability, EC materials can be exploited to develop electrochromic devices (ECDs), which are widely used for displays^3^, sensors^4^, energy storage devices^5^, and smart windows^6,7^. The EC materials are mainly categorised as inorganic or organic. Inorganic materials include transition metal oxides (e.g., tungsten oxide^8^, nickel oxide^9^) and inorganic complexes (e.g., organic framework^10^). Organic materials consist of π-conjugated organic molecules (e.g., viologen^11^), conductive polymers (e.g., polyimide^12^, polythiophene^13^), etc. Inorganic EC materials offer several advantages over organic ones, including high chemical stability and efficiency, as well as a memory effect after removing the external voltage, which are significant factors governing ECD applications^1^.

Among the existing EC materials in ECDs, those that switch between transparent and blue-tinted states have already been commercialised^14^, but grey or black tints are now required to meet the recent demand for darker states for both design reasons and to reduce environmental impact. If such material systems can be realised, they can be used as a window material in next-generation vehicles, such as electric vehicles and fuel cell vehicles, which are expected to become more popular in the future. When applied in windows, these materials can decrease electricity costs and increase the cruising range of these vehicles by reducing the air conditioning load.

To date, several studies have demonstrated black EC materials. However, many of these reported materials remain in the basic research stage because they are organic^15–18^, require a variety of materials for synthesis, are complex and time-consuming to process, and in some cases have a large environmental impact. Therefore, EC material systems that can express black in a simpler way are required.

Our group has been developing inks based on aqueous dispersions of tungsten oxide (WO_3_) and PB nanoparticles (NPs) for application in wet processes to prepare EC thin films^8,10^. Among wet processes, printing^19^ and coating^20,21^ offer the advantage of enabling EC thin film fabrication on large-scale substrates in a short time and at a low cost. In addition, these techniques are advantageous for thin film preparation on glass substrates and flexible substrates.

In this study, we developed a simple method for preparing dispersed suspensions of CuO NPs using basic copper(II) carbonate as the starting material and citric acid to adjust the pH of the water. Although methods for producing nanosized CuO particles have been extensively investigated^22–25^, there has been very little research on the preparation of stable, dispersed CuO NP suspensions, which are essential liquid-phase coatings for thin film production^26,27^. Biomedical applications of CuO NPs, for example, as sensor materials, glucose sensors, H2O2 sensors, dopamine sensors, and wound healing are well reported^28^. Colloidal suspensions containing oxide NPs have significant potential for application on an industrial scale, because they are easy to apply in continuous production processes, such as the printing and coating of nanostructured films^29^. Several attempts have been made to prepare CuO colloidal suspensions using nitrate- and acetate-based solutions^26,30–32^.

1 $$Cu{\left(N{O}_{3}\right)}_{2}\cdot 3{H}_{2}O+2NaOH\to Cu{\left(OH\right)}_{2}+2NaN{O}_{3}+3{H}_{2}O$$

2 $$Cu\left(C{H}_{3}COO\right)+2NaOH\to Cu{\left(OH\right)}_{2}+2Na(C{H}_{3}COO)$$

However, these processes are toxic to humans and require careful handling. They are also difficult to implement commercially when complex synthesis methods are required. Thus, the effective and cost-effective production of enough products within a given period to make these processes economically viable is a significant challenge.

**Figure 1:**
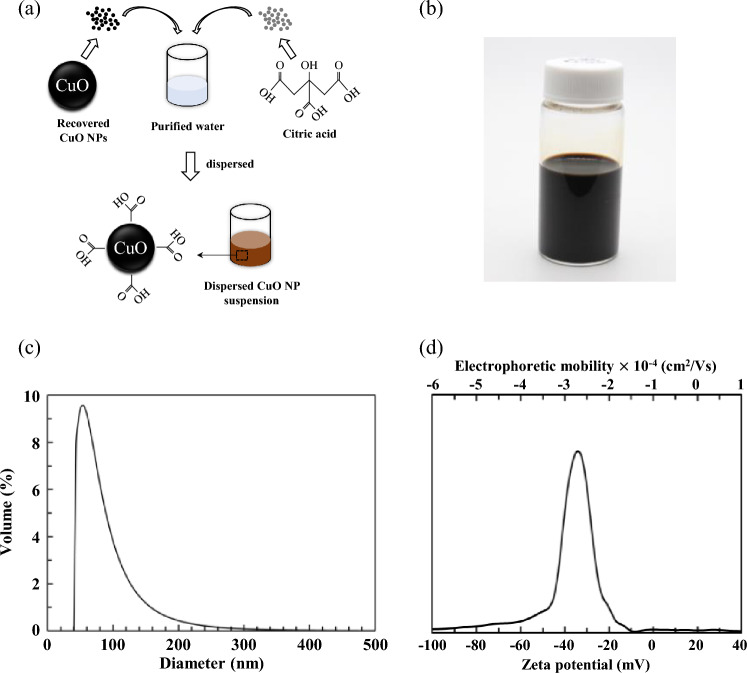
(**a**) Schematic of dispersed CuO NP suspension preparation and (**b**) photograph of prepared CuO suspension. (**c**) Mean (volume) diameter of the prepared CuO NP dispersed suspensions. (**d**) Zeta potential of the CuO suspensions.

In contrast to these previously reported methods, our process used no other materials like sodium hydroxide. In general, pure CuO particles do not disperse in water. Therefore, the surface of CuO was modified by adding citric acid to improve the water dispersibility (Fig. 1a). The NPs and dispersions of iron oxide, silver, and copper oxide have been prepared using citric acid in several studies, but they used materials that require careful handling, such as NaOH and metal nitrite^33–35^. There has been no report of the successful preparation and dispersion of such particles using citric acid alone thus far. In addition, CuO thin films are typically prepared by several different conventional methods, such as electrodeposition^36^, sol–gel^12^, and sputtering^37^. However, these methods are time-consuming and cannot be used to easily manufacture a large film. Therefore, some researchers have attempted to prepare large thin films in a short time at a low cost by coating or printing dispersed NP suspensions. In this study, we evaluated the applicability of a dispersed CuO NP suspension for coating functional thin films.

Furthermore, although CuO thin films offer many applications, such as batteries, gas sensors, and solar cells, this study was focused on their EC behaviour, which is a functional property of CuO. Richardson et al.^38^ observed that CuO thin films prepared by sputtering appeared dark in the coloured state. Therefore, the development of a coating method that can be used to easily fabricate large-size CuO has tremendous potential to replace conventional black organic EC materials^16,39–42^.

To that end, we used a dispersed CuO NP suspension to coat a functional thin film on an indium tin oxide (ITO)-coated glass substrate. Additionally, polyvinyl alcohol (PVA) was used to improve the adhesion of the CuO NPs to the ITO substrate in the thin films. The prepared CuO thin film exhibited a large change in the transmittance of 70% ↔ 6% at a wavelength of 633 nm via an electrochemical redox reaction, suggesting the possibility of producing a black material as an inorganic material. When understanding electrochemical behaviour and EC properties as one of the functionalities of CuO thin film, the treatment of the reaction mechanism and other aspects of the electrochemical behaviour is complicated due to the existence of various electrolytes and other cell structures. In order to use CuO as a stable black electrochromic material, it was assumed that it would be difficult to analyze the detailed reaction mechanism in thin films prepared by existing methods. However, the synthesis method of CuO-based nanoparticle suspensions discovered in this study enabled us to proceed with a detailed mechanism analysis of the electrochromic properties of the resulting CuO thin films.

## Results

### Formation of dispersed CuO NP suspension

The surface tension and viscosity of the dispersed CuO NP suspension synthesized by the procedure in Fig. 1a were 69 mN/m and 1.19 cP, respectively. A photograph of the suspension is shown in Fig. 1b. The density and pH of the suspension were 4.67 g/cm^3^ and 6.0, respectively.

The volume mean diameter of the particles in the synthesised CuO NP suspensions was determined to be 56 nm using dynamic light scattering (DLS) measurements (Fig. 1c). In addition, the zeta potential was measured to analyse the stability of the CuO suspension, which indicates the possible behaviour of the dispersion. The CuO suspensions under three different PVA conditions are shown in Fig. 1d. A mean zeta potential of − 34.9 mV was obtained for the suspensions; zeta potential values between − 30 and − 40 mV typically indicate moderate stability^43,44^. For comparison, colloidal solutions were prepared with 95.0% and 99.9% copper(II) oxide, which are commercially available, under the same conditions used to synthesise the CuO NPs. However, these powders did not disperse well in water containing dissolved citric acid (Supplementary Fig. S1).

### Structure and chemical composition of the CuO nanoparticles and films

**Figure 2:**
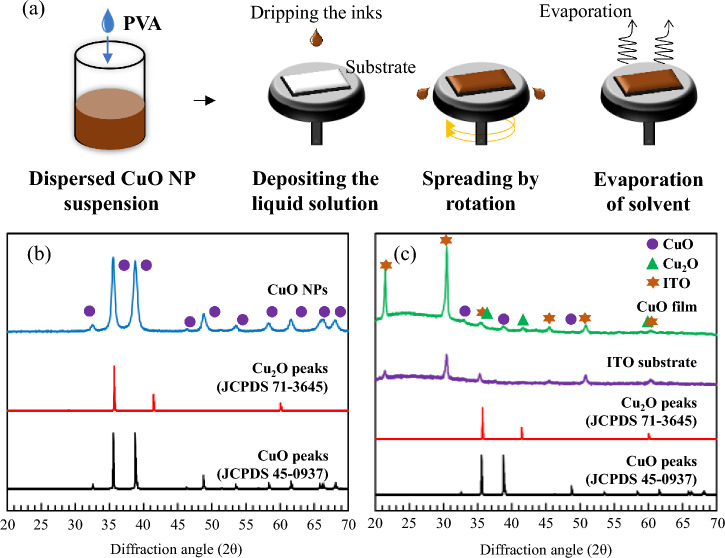
(**a**) Schematic of CuO film preparation. (**b**) XRD patterns of nanopowders obtained from solvent-evaporated dispersed CuO NP suspensions and (**c**) CuO thin films coated on ITO substrates.

To determine the crystal structure and phase composition of the CuO NPs obtained after solvent evaporation at 120 °C in a dry oven and the CuO film prepared by the spin-coating method (Fig. 2a), X-ray diffraction (XRD) patterns were recorded for all synthesised samples, as shown in Fig. 2b. The XRD patterns of the CuO nanopowders were identical to that of monoclinic single-phase CuO, and the diffraction data corresponded well with that of the CuO JCPDS card (JCPDS 45-0937), with no impurity peaks present. Furthermore, the primary crystallite size (~ 20 nm) determined by the Pawley method, combined with the results of XRD analysis, confirmed the material dispersed in the suspension to be CuO NPs. Interestingly, a (220) peak, corresponding to Cu_2_O (JCPDS 71-3645), was observed at ~ 42° for the spin-coated films, as shown in Fig. 2c. This peak may indicate that Cu_2_O is formed in the outer layers of the film owing to the partial reduction of Cu(II) to Cu(I).

**Figure 3:**
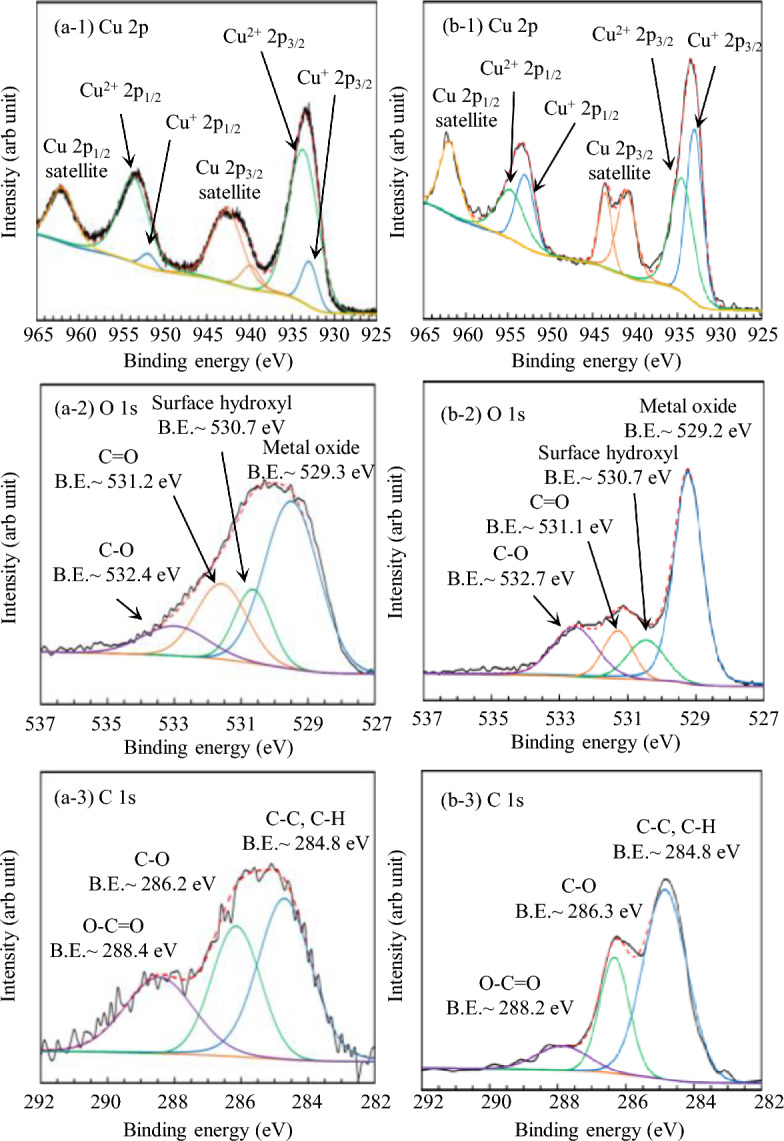
High-resolution XPS profiles of (**a**) nanopowders obtained from solvent-evaporated dispersed CuO NP suspensions and (**b**) CuO thin films prepared on ITO substrates.

X-ray photoelectron spectroscopy (XPS) was employed to investigate the chemical composition of the CuO NP powders and CuO films prepared on the ITO substrates (Supplementary Table S1). The XPS survey spectra of the CuO films showed no impurity peaks, and exhibited only the peaks corresponding to Cu, O, and C. Figures 3a-1, b-1 show the high-resolution spectra of Cu 2p, which are separated into the Cu 2p_3/2_ and Cu 2p_1/2_ peaks observed at approximately 932.3 ± 0.1 eV and 952.2 ± 0.3 eV, respectively^45,46^. The distance between these Cu 2p main peaks was 19.9 eV, which agreed with that reported for the CuO spectrum^47^. Moreover, Fig. 3a-1, b-1 clearly show that the Cu 2p spectra contain “shake-up” satellites, which are characteristic of the Cu^2+^ state^45^. These satellite peaks are deviated by ~ 9 eV from the main peak (the binding energies (E_*b*_) are approximately 941 and 962 eV for Cu 2p_3/2_ and Cu 2p_1/2_, respectively). Intense peaks corresponding to Cu^2+^ 2p_3/2_ and Cu^2+^ 2p_1/2_ were observed for the CuO NPs, indicating that the prepared NP sample contained CuO (Fig. 3a-1). In contrast, intense Cu^+^ 2p_3/2_ and Cu^+^ 2p_1/2_ peaks were observed for the CuO film prepared on the ITO substrate. This indicates that the film primarily contains CuO, and the outer layer contains Cu_2_O, in which Cu(II) partially oxidised to Cu(I)^48^. This was in agreement with the XRD results. The O 1s XPS profiles are presented in Fig. 3a-2, b-2. A clear peak is observed at 530.3 ± 0.2 eV, which can be indexed to O^2−^ in CuO^49^. Notably, three other weak O 1s peaks are also present. The peak located at 531.7 ± 0.2 eV originates due to surface hydroxyls^49,50^, while the peaks at 532.5 and 533.8 eV correspond to C=O and C–O, respectively^51^. The latter peaks can be attributed to the citric acid and PVA used in the preparation of the dispersed CuO NP suspension. The C 1s signal of the CuO films (284.8 eV) in Fig. 3a-3, b-3 can be attributed to the C–C bonds of PVA and citric acid^48,52^.

To investigate the effect of PVA addition on the film-forming characteristics of CuO NP suspensions, field-emission scanning electron microscopy (FESEM) was used to study their morphological features. Supplementary Fig. S2 shows the surface and cross-sectional FESEM images of 1 wt.% PVA CuO films. From the cross-sectional image shown in Supplementary Fig. S2a, the CuO films are approximately 200 nm thick and exhibit good adhesion between the ITO substrates and CuO coatings. Furthermore, the surface morphology seen in Supplementary Fig. S2b indicates that the film exhibits a porous structure. This porous structure could enhance the Li^+^ diffusion in the film and contribute to its cationic conductivity^53^.

### Electrochemical and electrochromic properties

**Figure 4:**
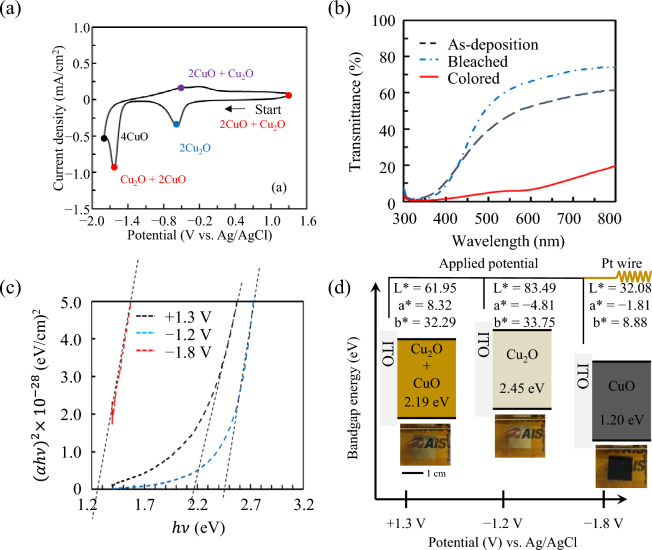
Electrochemical and EC properties of the CuO films in LiClO_4_/PC (1 mol/kg) electrolyte solution. (**a**) CV profiles of CuO thin films. The potential scan rate was 5 mV/s. (**b**) Bleached (blue line), coloured (red line), and as-prepared (black line) transmittance spectra of CuO thin films. Constant voltages of –1.8 and + 1.3 V (vs. Ag/AgCl) were applied for 30 s each to colour and bleach the films, respectively. (**c**) ${(\alpha h\nu )}^{2}$ *vs.* photon energy for CuO thin films in as-prepared, coloured at –1.8 V and bleached at + 1.3 V states. Intercepts of the dashed lines correspond to the direct band gaps. (**d**) Schematic representation of the energy band diagram and photos of the CuO composite.

The electrochemical and EC properties of the CuO films deposited on the ITO substrates were analysed using a three-electrode chemical cell containing aqueous LiClO_4_/PC (1 mol/kg) as the electrolyte. The cyclic voltammetry (CV) measurements were conducted in the potential range from − 1.8 to + 1.3 V vs. Ag/AgCl at a sweep rate of 5 mV/s. The results are shown in Fig. 4a and Supplementary Fig. S3. The CuO film exhibits a complex redox reaction, and we propose the following four steps of electrochemical reaction mechanism. (1) The prepared thin film is in a state in which CuO and Cu_2_O are a mixture at the beginning of the reaction. (2) A cathodic peak^54^, which corresponds to Cu_2_O + 2CuO + 2Li^+^  + 2e^−^  → 2Cu_2_O + Li_2_O^55–57^, appeared at approximately − 0.5 V, and the transmittance increased slightly to − 1.2 V. This is attributed to the reduction of CuO present in the thin film to Cu_2_O, and is consistent with the XPS results described above. (3) Furthermore, an oxidation reaction, which corresponding to 2Cu_2_O + 2Li_2_O → 4CuO + 4Li^+^  + 4e^−56^, was initiated at approximately − 1.8 V, which caused the film colour to change to dark. A peak corresponding to CuO was observed in the XPS results of the black CuO thin film (Supplementary Fig. S4), and it is considered that Cu_2_O^58^ and Li_2_O^59^ did not fully participate. Finally, (4) it is thought that the film becomes CuO state due to 4CuO + 2Li^+^  + 2e^−^  → 2CuO + Cu_2_O + Li_2_O at approximately − 0.2 V. The transferred charge density (Δ*Q*), which indicates the electrochemical activity of the CuO film, was determined by performing chronocoulometry (CC) at constant applied potentials of − 1.8 and + 1.3 V (vs. Ag/AgCl) for 90, 120, and 120 s, respectively, to allow sufficient time for the complete redox reaction in each film (Supplementary Fig. S5). A Δ*Q* value of 68 mC/cm^2^ was observed for the CuO film.

Changes in the optical transmittance spectra of the as-deposited, transparent brown, and dark grey colour states of the CuO films were studied using ultraviolet–visible (UV–Vis) spectroscopy during CC analysis, as shown in Fig. 4b. The change in the transmittance of the CuO film with a thickness of 200 nm at + 1.3 and − 1.8 V was approximately 63% at a wavelength of 633 nm. However, the transmittance of the 150-nm-thick film in the coloured state increased, and the transmittance of the 300-nm-thick film in the bleached state decreased (Supplementary Fig. S6). Therefore, the film thickness of 200 nm was considered the most appropriate for application as an EC material. Moreover, the optical density (Δ*OD*) was used to calculate the EC colouration efficiency (CE), which is an important EC parameter that determines the electrochemical performance of the films (Supplementary Table S2). This parameter is defined in Eqs. (3) and (4):

3 $$CE= \frac{\Delta OD}{\Delta Q}$$

4 $$\Delta OD(\lambda )={\mathrm{log}}_{10}({T}_{\lambda b}/{T}_{\lambda c})$$

where *λ* = 633 nm, and *T*_*λb*_ and *T*_*λc*_ represent the transmittance of the bleached and coloured states, respectively. The Δ*OD* and CE values obtained for the CuO films were 1.04 and 15.36 cm^2^/C, respectively.

The band gaps (*E*_*g*_) of the films in the as-deposited, transparent brown, and dark grey states can be estimated using the transmittance data and estimated film thickness (*d* = 200 nm). Figure 4c shows a graphical representation of (*αhv*)^2^ vs. photon energy (*hv*) for these states, which can be evaluated using the following equation^54,60,61^:

5 $$\alpha =\frac{1}{d}ln\frac{100}{T}$$

where *α*, *d*, and *T* are the absorption coefficient, film thickness, and transmittance value, respectively. The optical density can be used to obtain the band gap energy of the transparent films by plotting (*αhν*)^1/η^ against *hν*, based on the following relation^58–61^:

6 $$\alpha h\nu =A{(h\nu -{E}_{g})}^{\eta }$$

where *A* and *η* are constants, and *η* depends on the nature of the transition. For copper-oxide-based materials, it is assumed that *η* = ½, which corresponds to a directly allowed electron transition mechanism^48,50,52^. Therefore, in the initial absorption region (*hν* ≈ *E*_g_), where the plot of (α*h*ν)^2^ vs. *h*ν is linear, the intercept of the extrapolated fitted line with the *h*ν axis gives the optical band gap energy, as shown in Fig. 4c. Direct optical band gap energies for the Cu_2_O and CuO films, which have been reported to be in relatively wide ranges of approximately 2.1–2.6 eV and 1.3–1.7 eV, respectively^54,62–67^ are dependent on the fabrication method and stoichiometry. The experimentally obtained optical band gap values were compared with the reported data, as shown in Fig. 4d. The transmittance in the as-deposited state, the transparent brown state, and the dark grey state corresponded to the Cu_2_O + CuO compound film, Cu_2_O, and CuO + Cu films, respectively. The colour details were also evaluated based on the band gap change using the International Commission on Illumination (CIE: usually abbreviated CIE for its French name, Commission Internationale de l'Eclairage) 1976 L*a*b* colour model. The L* and b* values decreased from 83 to 32 and from 34 to 9, respectively, and the a* value increased from − 5 to − 2 as Cu_2_O was oxidised to CuO, which is indicative of a nearly dark grey colour (Supplementary Fig. S7). Several groups previously reported the chromaticity of dark organic EC materials prepared by different techniques. For instance, Wu et al.^68^ observed that the L* values of amine-based organic EC materials decreased from 92 to 6 upon changing from the bleached to the coloured state. Further, Liu et al.^42^ also reported a change in L* between 88 and 7 with an organic EC device composed of polyamide and viologen.

Here, we summarise the superiority of the results of this study by comparing them with those of existing studies. Although organic materials exhibit excellent EC properties, they have several disadvantages, including the use of harmful substances and complicated manufacturing processes, as well as poor durability against external stimuli (UV, temperature, etc.). In contrast, the CuO ink used in this study can be synthesised simply by mixing basic copper(II) carbonate with citric acid, which enabled coating CuO thin films. Furthermore, the CuO thin film prepared in this study showed better EC properties than any previously reported CuO thin films in Li-based electrolytes^54^ and showed low transmittance at visible wavelengths of 380–780 nm, which is comparable to that of organic EC materials. Moreover, as a nanomaterial, the CuO ink in this study has many potential applications at the cutting edge of science and technology, including catalysis^69^, gas sensors^70^, photovoltaic cells^71^, light-emitting diodes^72^, magnetic phase transitions^73^, and superconductors^74^.

## Discussion

We developed a facile method for synthesising dispersed CuO NP suspensions using only citric acid, basic copper(II) carbonate, and water. This method does not require conventional toxic chemicals such as metal nitrite or NaOH and significantly reduces the suspension preparation time and cost. The PVA was added to the suspensions to help evaluate the ability to deposit CuO thin films on an ITO substrate by spin coating, which is an inexpensive and simple film preparation method. The XRD results indicated strong CuO peaks for the powder obtained from the suspensions; however, Cu_2_O (200) peaks appeared for the spin-coated films. This may indicate the formation of Cu(II) in the outer layers of the film owing to the partial oxidation of Cu(I), which is consistent with the XPS results. The CV profiles displayed redox peaks, which corresponded to the reversible transition between Cu_2_O and CuO. The films also exhibited EC behaviour during the redox reaction; the colour changed to grey and transparent brown at negative and positive voltages, respectively. The difference in transmittance values between the grey and transparent film states at 633 nm was ~ 40%. The band gaps for the three film states (as-prepared, dark grey, and transparent brown) were determined from the transmittance measurements, based on a direct semiconductor transition mechanism. These results confirmed that the EC process was driven by the conversion of Cu_2_O to CuO in a reversible redox reaction on the film surface. Because this material is suggested to be superior to organic black EC materials in terms of lightfastness (life span), environmental testing is scheduled to demonstrate its practical feasibility. In particular, in an ongoing research project (JST A-STEP (Grant Number JPMJTR203D)), we are conducting research and development of black EC devices that can be mounted on automobile window glass in collaboration with a company. Although there are many materials and principles of blue and transparent colour change in commercial EC devices, since blue tones have compatibility and affinity with body colours when installed in automobiles, "blackness" is one of the important development elements not only in terms of mere functionality, but also in terms of design. Furthermore, in Japan, for example, there is a large temperature difference between the four seasons (−20 °C in winter and nearly 40 °C in summer), and the rainy season is a very harsh natural environment. Therefore, based on the results obtained in this study, the company plans to convert to a stable deposition process (large area and uniformity) and advance the durability (light resistance, heat and humidity resistance, etc.) required for in-vehicle applications. The results obtained in terms of durability and other aspects, as well as approaches to overcoming issues, will be reported continuously in the future. Finally, although the main research objective of this study was to create black EC materials, the CuO NP dispersion suspensions produced by this extremely simple method could be applied in dye-sensitised solar cells, gas sensors, batteries, and EC devices using other coating technologies such as inkjet and slit coating.

## Methods

### Reagents

The following reagents were purchased and used without further purification: basic copper(II) carbonate (Cu_2_CO_3_(OH)_2_; FUJIFILM Wako Pure Chemical Co.), citric acid (C_6_H_8_O_7_; FUJIFILM Wako Pure Chemical Co.), polyvinyl alcohol (PVA; 99%; Japan Vam & Poval Co., Ltd.), and lithium perchlorate (LiClO_4_; FUJIFILM Wako Pure Chemical Co.). The ITO-coated glass with a surface resistivity of 10 Ω/sq was obtained from Geomatec.

### Synthesis of dispersed CuO nanoparticle suspension

The CuO NPs were obtained by thermally treating basic copper carbonate at 320 °C for 3 h using a muffle furnace (FP21, Yamato Scientific Co.) under atmospheric conditions^75^. A citric acid solution (2.0 M, pH: 3.0) was prepared using purified water before adding the CuO NPs. The pH of the citric acid solution was ~ 2.5. Next, the CuO NPs (20% of the total ink weight) were mixed in purified water by stirring for 24 h at 1000 rpm at room temperature (~ 20 °C). The CuO NPs were then recovered by centrifugal separation at 18,000*g* for 10 min. Subsequently, the CuO NPs were dispersed by adding pure water to prepare a CuO NP suspension. Regarding these synthesis methods, our research group has expertise in the synthesis of Prussian blue NP and follows that method^76,77^. The CuO content in the final suspension was maintained at 20% of the total weight. The process for synthesising the dispersed CuO NP suspensions is shown in Supplementary Fig. S8.

### Particle size and zeta potential

Since the synthesized CuO NP were expected to be quite small in this study, the volume-averaged diameter and zeta potential of the dispersed CuO NP suspension were measured by Dynamic Light Scattering (ELS-Z 2, Otsuka Electronics Co., Ltd.).

### Film preparation

The viscosity of the as-prepared suspension was low, making it difficult to prepare the CuO thin films. Thus, to improve the adhesion of the CuO NP suspension to the ITO glass substrate and increase its viscosity, 1 wt.% PVA was added. Prior to film preparation, a 25-cm^2^ ITO substrate was subjected to plasma treatment for 3 min using a plasma cleaner (PDC-001, Harrick Plasma Inc.) to increase its wettability towards the CuO suspension. Plasma processing was performed under a radio frequency (RF) glow discharge at a low pressure of 1 × 10^–3^ Torr and an applied wattage of 30 W. The CuO thin films were then deposited on the ITO substrates using a spin-coating machine (ACT-300AII, Active Inc.). To evaluate the characteristics of the film, CuO thin film samples (sizes: 2.5 cm^2^ for XRD and 1 cm^2^ for XPS/FESEM) were prepared after cutting them with a diamond blade.

### Film characterisation

The crystallinity of the CuO nanopowders was determined by first evaporating the solvent from the NP suspension at 120 °C in a dry oven. The powders were then analysed by XRD at 40 kV and 40 mA using Cu-Kα radiation (λ = 1.5418 Å) (D8 Advance, Bruker AXS Inc.); the data were collected within a diffraction angle (2θ) range of 20°–80°. The size of the primary crystallites was calculated with TOPAS V5.0 software using the Pawley method. The XPS (PHI 5000 Versaprobe; Ulvac-Phi, Inc.) was conducted with Al-Kα radiation. Survey scans were recorded in the range of 0–1000 eV, with a pass energy of 117.4 eV and resolution of 0.2 eV. High-resolution scans were obtained at a pass energy and resolution of 23.5 and 0.025 eV, respectively. To avoid sample charging, a neutraliser filament was used. Adventitious carbon, with a C 1s peak at 248.8 eV, was used to calibrate the spectra and correct for peak shifts due to charging. The obtained Cu 2p XPS profiles were deconvoluted using a nonlinear least-squares curve-fitting program (MultiPak Version 9.6 software), and the Shirley background was used for spectral deconvolution. The peaks were mixed (80% Gaussian and 20% Lorentzian) and constrained by a Cu 2p_3/2_–Cu 2p_1/2_ spin–orbit separation of 19.9 eV; the area ratio of the two peaks in each doublet was 0.75. Qualitative XPS scans of the CuO films were generated in the binding energy (*E*_b_) range of 925–970 eV. The cross-sectional morphologies of the films and their thicknesses were characterised by FESEM (S-4800; Hitachi, Ltd., Japan) with an accelerating voltage of 5 kV after coating the samples with Pt–Pd using an ion sputter coater (E-1030; Hitachi, Ltd., Japan).

The deposition density of the CuO suspension was calculated using Eq. (7) ^78^:

7 $$\rho =m / (A\times d)$$

where *d* is the film thickness, *m* is the weight of the coated dispersed CuO NP suspension, *A* is the area of the ITO substrate, and *ρ* is the suspension density. The *d* values of the CuO films were estimated by FESEM through the cross-sectional morphology of the films. A thickness of 200 nm was observed for the prepared CuO film, and the calculated *ρ* was 4.67 g/cm^3^.

### Electrochemical and electrochromic analysis

To investigate the electrochemical and EC properties of the films, CV and CC measurements were performed using aqueous LiClO_4_/PC (1.0 mol/kg), which is a polymer that contains a Li salt, in a conventional three-electrode cell with an electrochemical measurement system (6115D, ALS/HCH). A WO_3_ film deposited on the ITO substrate served as the working electrode; a Pt wire was used as the counter electrode, and Ag/AgCl was used as the reference electrode; LiClO_4_ was used as a supporting electrolyte. The in-situ transmittance was determined using a multichannel charge-coupled device (CCD) detector (DH-2000, Ocean Optics). The chromaticity of the 200-nm CuO film was measured using a spectrophotometer (SD 3000, Nippon Denshoku Industries Co. Ltd.).

## Supplementary Information

Supplementary Information.

## Data Availability

The data supporting the findings of this study are available from the corresponding author upon reasonable request.
