# Supplementary material for: Black electrochromic ink with a straightforward method using copper oxide nanoparticle suspension
Source: Sci Rep. 2023 May 13;13:7774. doi: 10.1038/s41598-023-34839-9 (PMC10182978; doi:10.1038/s41598-023-34839-9)
Supplement: Supplementary file 1 — Supplementary Information. [file 41598_2023_34839_MOESM1_ESM.pptx]

## Slide 1
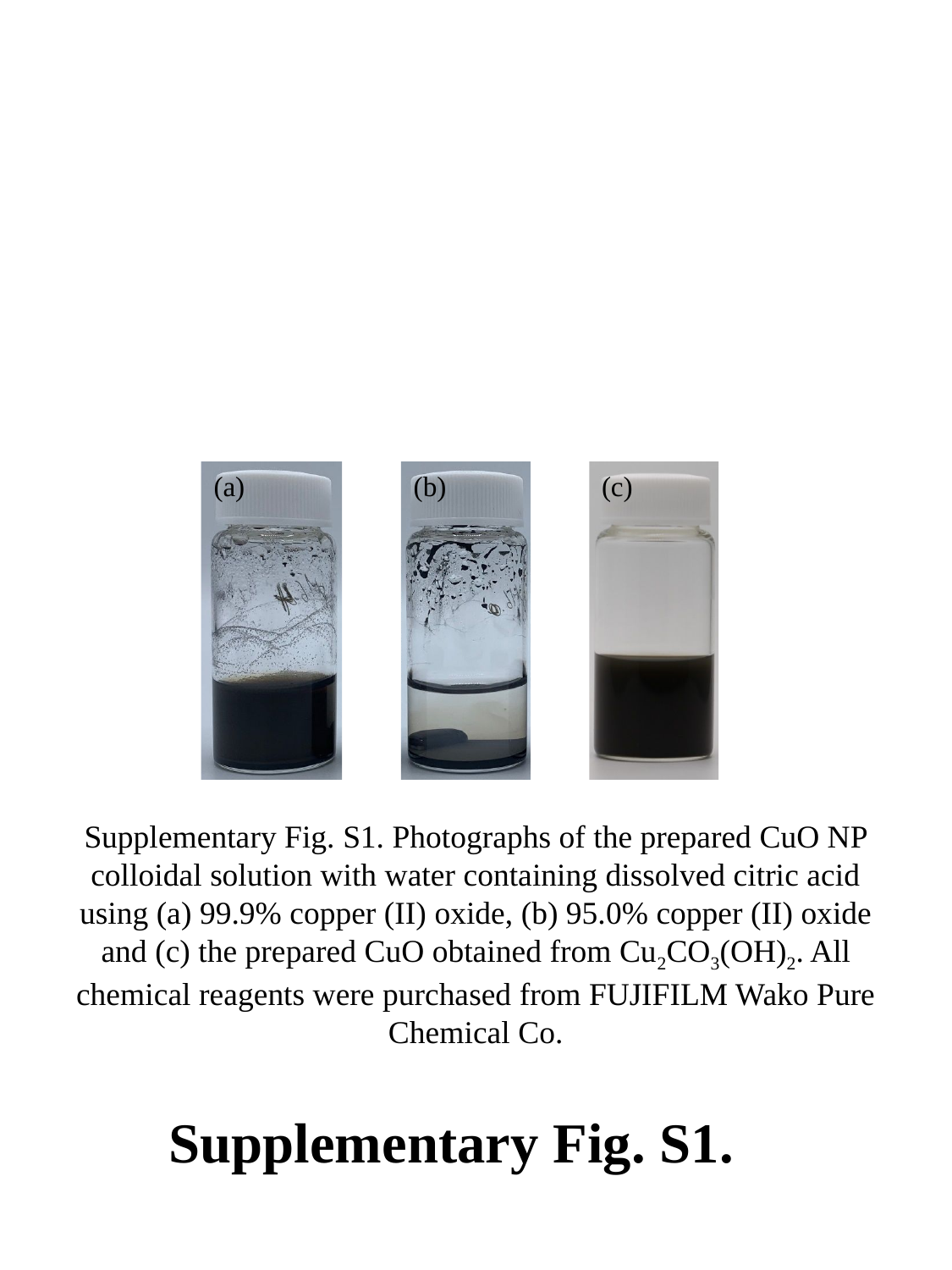

(a)
(b)
(c)
Supplementary Fig. S1. Photographs of the prepared CuO NP colloidal solution with water containing dissolved citric acid using (a) 99.9% copper (II) oxide, (b) 95.0% copper (II) oxide and (c) the prepared CuO obtained from Cu2CO3(OH)2. All chemical reagents were purchased from FUJIFILM Wako Pure Chemical Co.
Supplementary Fig. S1.

## Slide 2
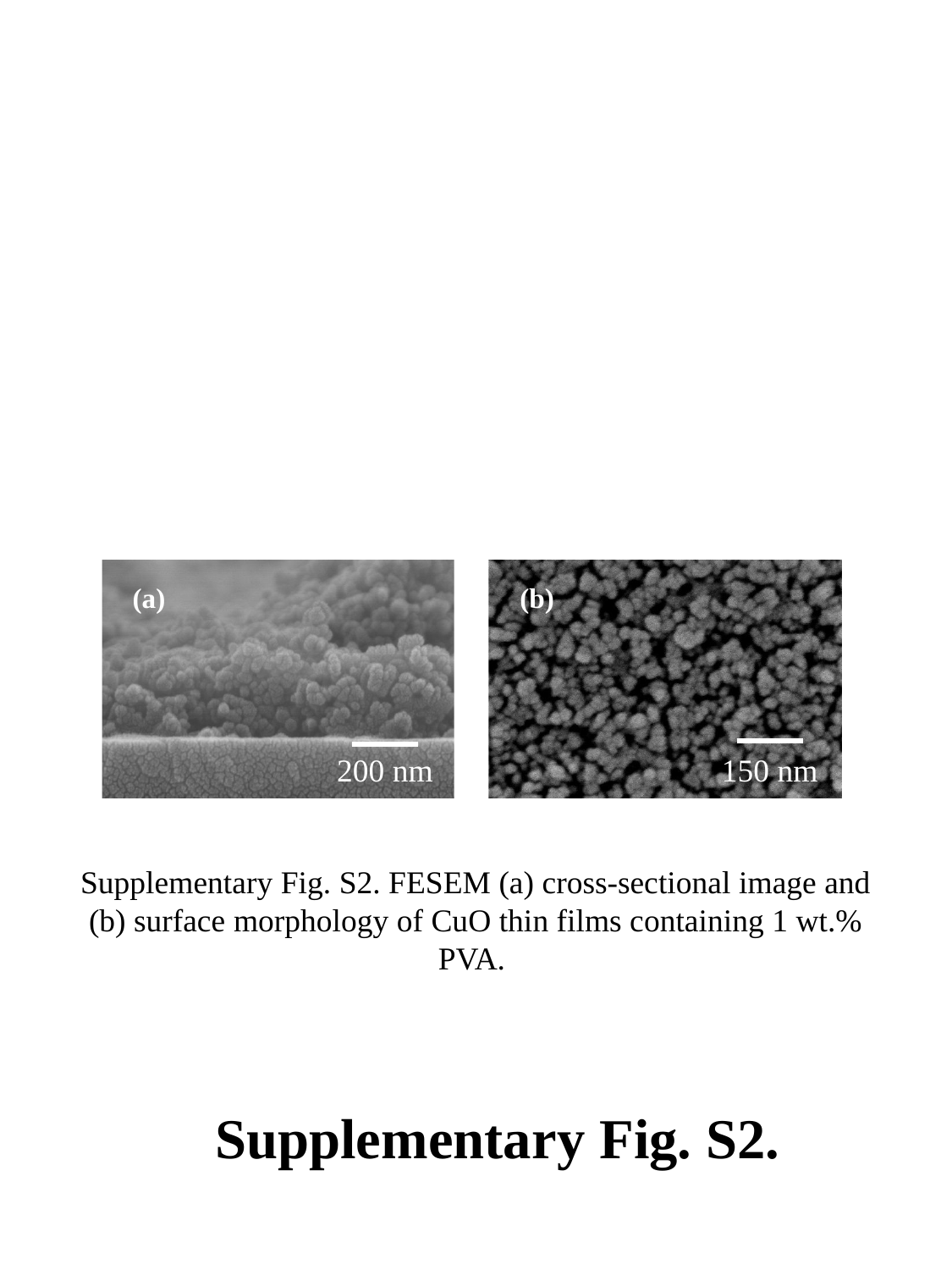

(a)
(b)
200 nm
150 nm
Supplementary Fig. S2. FESEM (a) cross-sectional image and (b) surface morphology of CuO thin films containing 1 wt.% PVA.
Supplementary Fig. S2.

## Slide 3
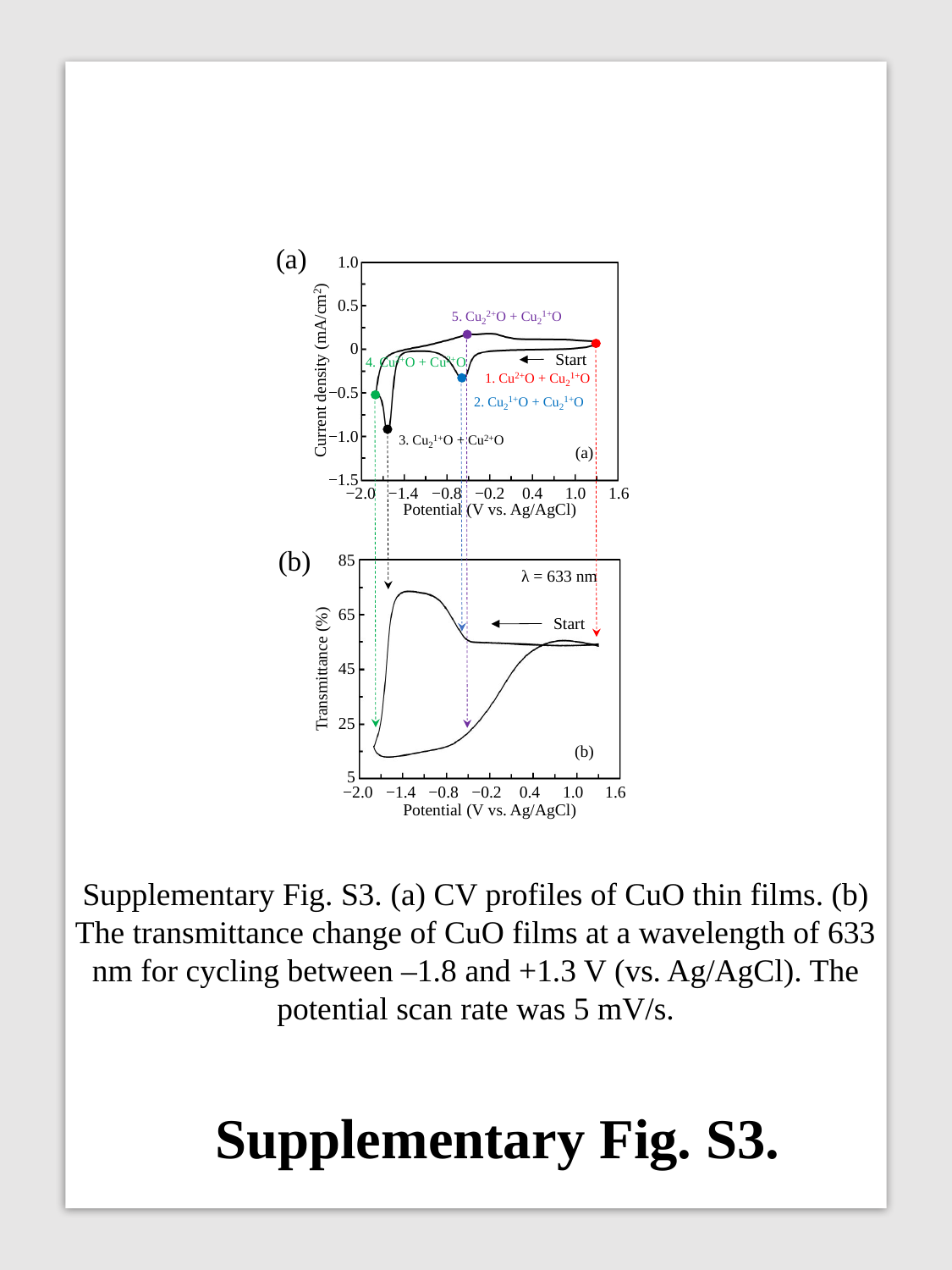

(a)
(b)
Supplementary Fig. S3. (a) CV profiles of CuO thin films. (b) The transmittance change of CuO films at a wavelength of 633 nm for cycling between –1.8 and +1.3 V (vs. Ag/AgCl). The potential scan rate was 5 mV/s.
Supplementary Fig. S3.

## Slide 4
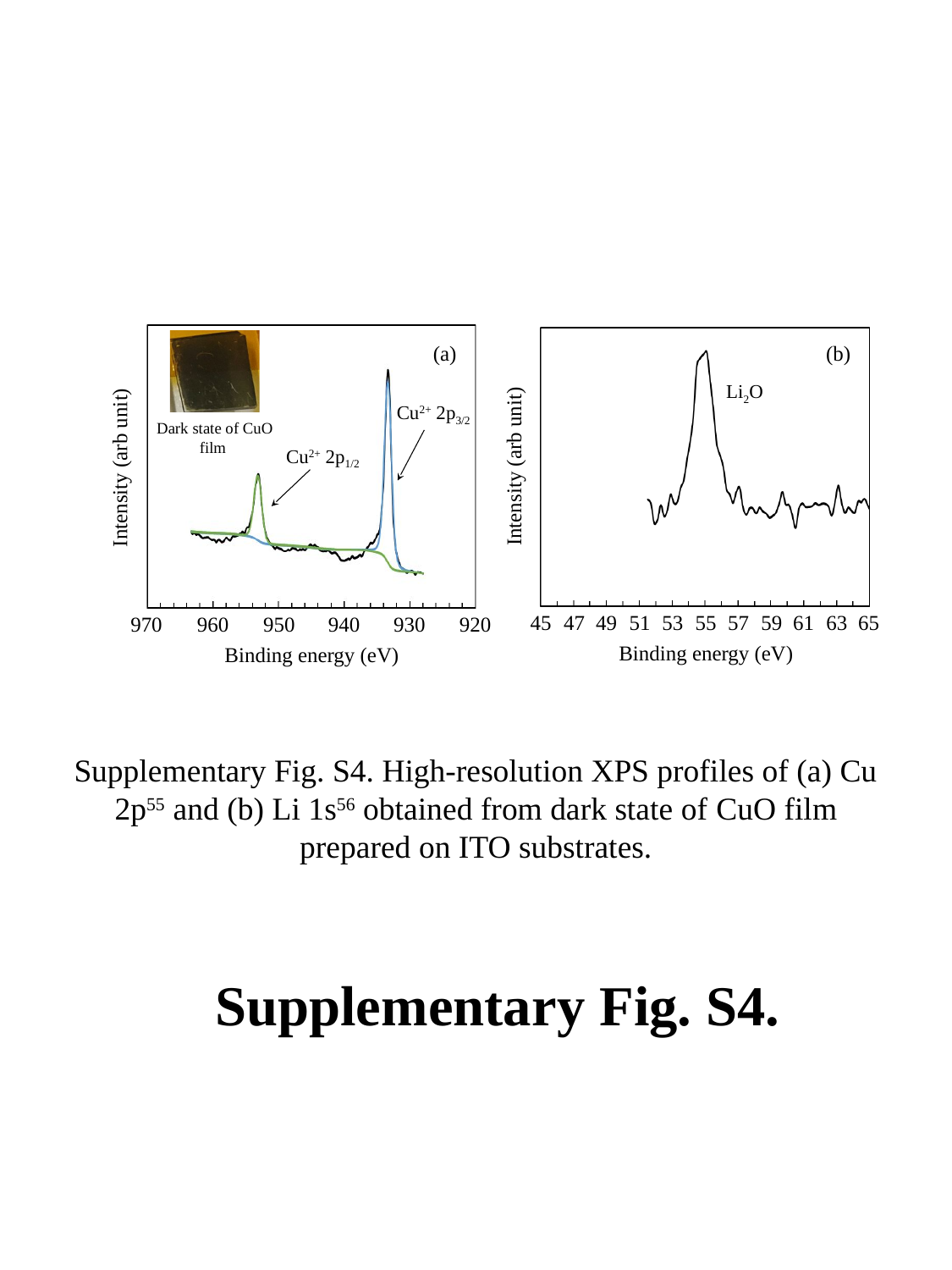

(a)
(b)
Li2O
Cu2+ 2p3/2
Dark state of CuO film
Cu2+ 2p1/2
Intensity (arb unit)
Intensity (arb unit)
45
47
49
51
53
55
57
59
61
63
65
970
960
950
940
930
920
Binding energy (eV)
Binding energy (eV)
Supplementary Fig. S4. High-resolution XPS profiles of (a) Cu 2p55 and (b) Li 1s56 obtained from dark state of CuO film prepared on ITO substrates.
Supplementary Fig. S4.

## Slide 5
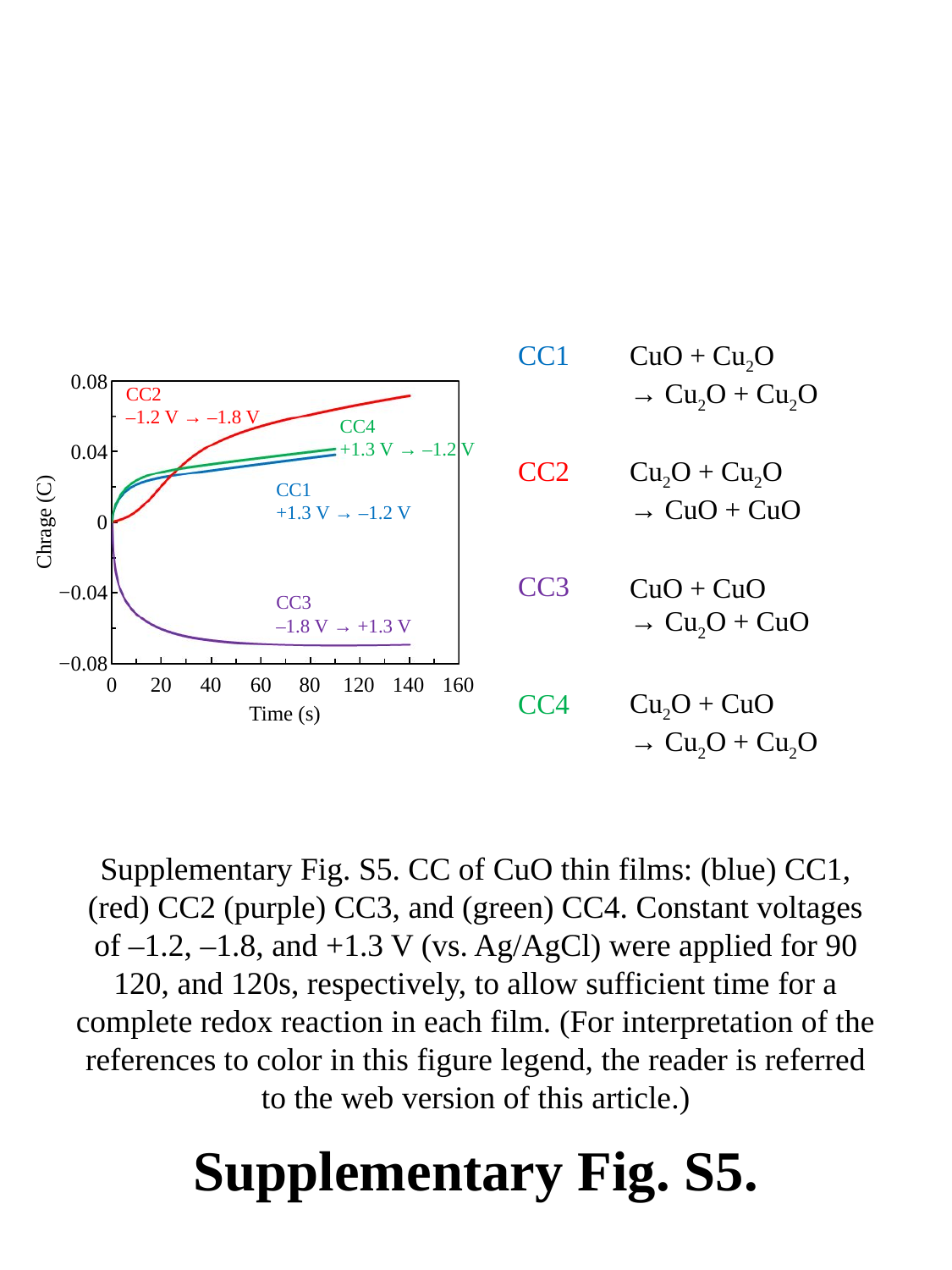

CC1
CuO + Cu2O
→ Cu2O + Cu2O
0.08
CC2
–1.2 V → –1.8 V
CC4
+1.3 V → –1.2 V
0.04
CC2
Cu2O + Cu2O → CuO + CuO
CC1
+1.3 V → –1.2 V
0
Chrage (C)
CC3
CuO + CuO
→ Cu2O + CuO
−0.04
CC3
–1.8 V → +1.3 V
−0.08
0
20
40
60
80
120
140
160
Cu2O + CuO
→ Cu2O + Cu2O
CC4
Time (s)
Supplementary Fig. S5. CC of CuO thin films: (blue) CC1, (red) CC2 (purple) CC3, and (green) CC4. Constant voltages of –1.2, –1.8, and +1.3 V (vs. Ag/AgCl) were applied for 90 120, and 120s, respectively, to allow sufficient time for a complete redox reaction in each film. (For interpretation of the references to color in this figure legend, the reader is referred to the web version of this article.)
Supplementary Fig. S5.

## Slide 6
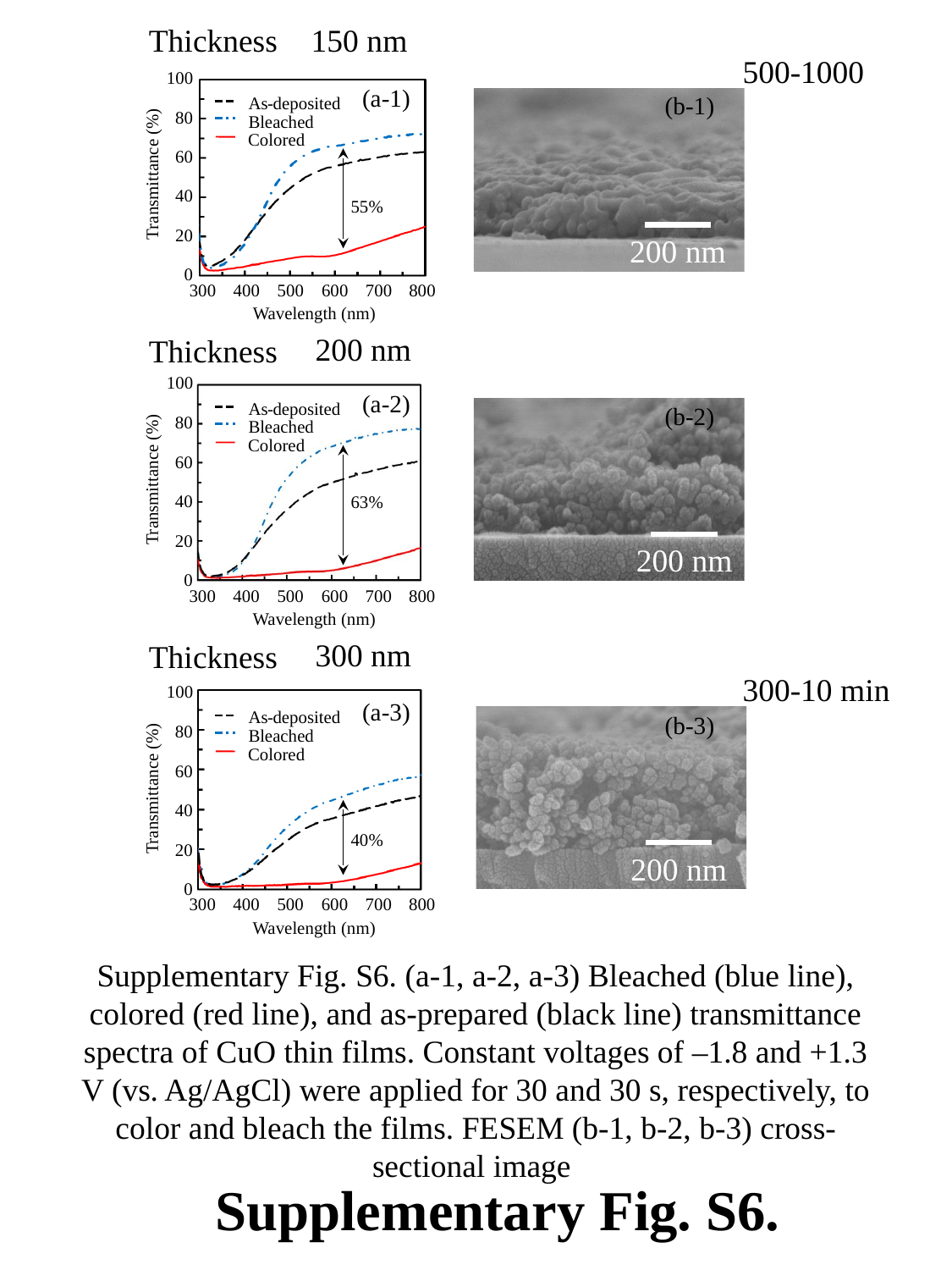

Thickness
150 nm
500-1000
100
(a-1)
(b-1)
As
-
deposited
80
Bleached
Colored
60
Transmittance (%)
40
55%
20
200 nm
0
300
400
500
600
700
800
Wavelength (nm)
200 nm
Thickness
100
(a-2)
(b-2)
As
-
deposited
80
Bleached
Colored
60
Transmittance (%)
63%
40
20
200 nm
0
300
400
500
600
700
800
Wavelength (nm)
300 nm
Thickness
300-10 min
100
(a-3)
(b-3)
As
-
deposited
80
Bleached
Colored
60
Transmittance (%)
40
40%
20
200 nm
0
300
400
500
600
700
800
Wavelength (nm)
Supplementary Fig. S6. (a-1, a-2, a-3) Bleached (blue line), colored (red line), and as-prepared (black line) transmittance spectra of CuO thin films. Constant voltages of –1.8 and +1.3 V (vs. Ag/AgCl) were applied for 30 and 30 s, respectively, to color and bleach the films. FESEM (b-1, b-2, b-3) cross-sectional image
Supplementary Fig. S6.

## Slide 7
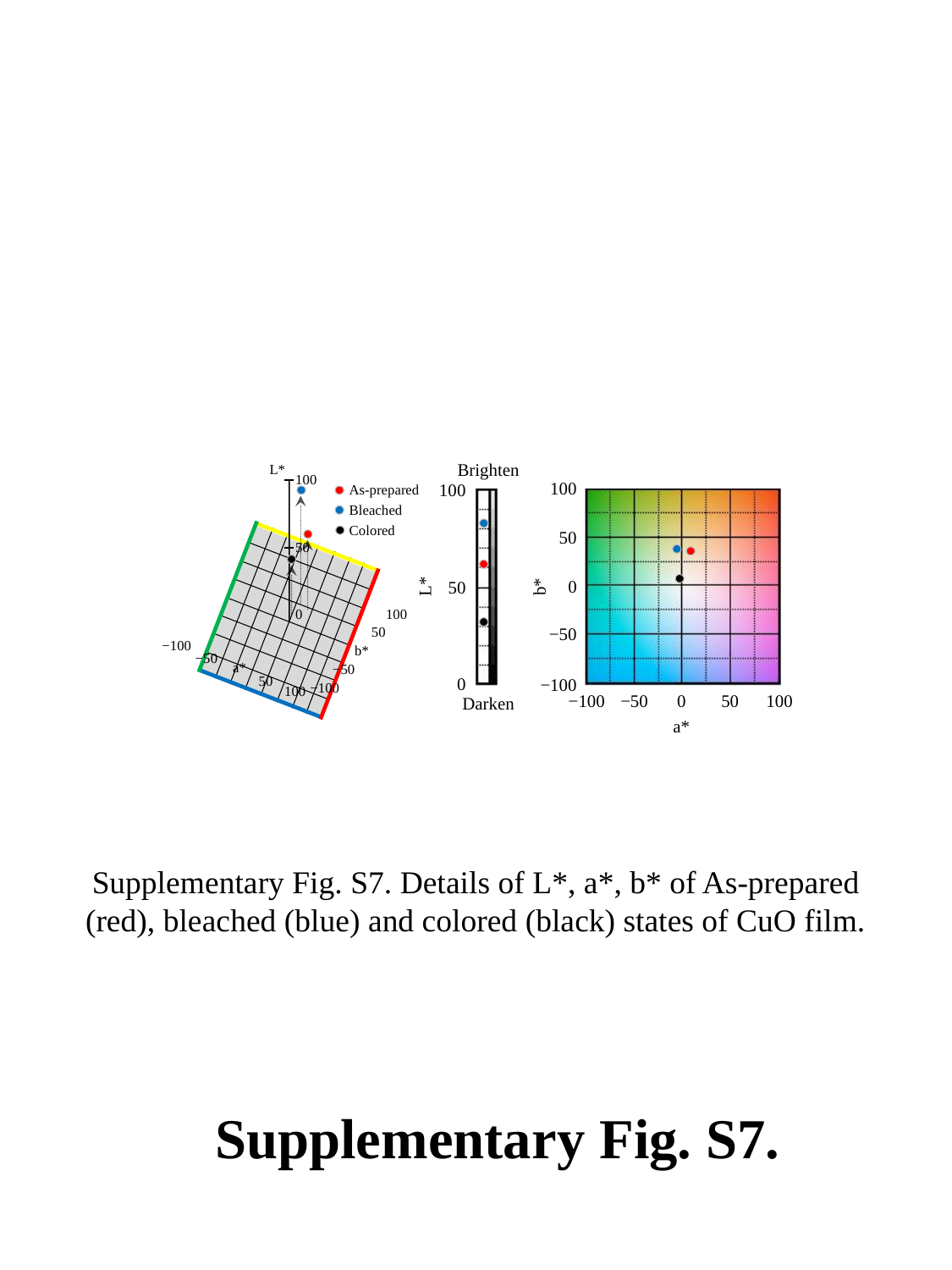

Brighten
L*
100
100
100
As-prepared
Bleached
Colored
50
50
0
L*
b*
50
0
100
−50
50
−100
b*
−50
a*
−50
50
0
−100
−100
100
−100
−50
0
50
100
Darken
a*
Supplementary Fig. S7. Details of L*, a*, b* of As-prepared (red), bleached (blue) and colored (black) states of CuO film.
Supplementary Fig. S7.

## Slide 8
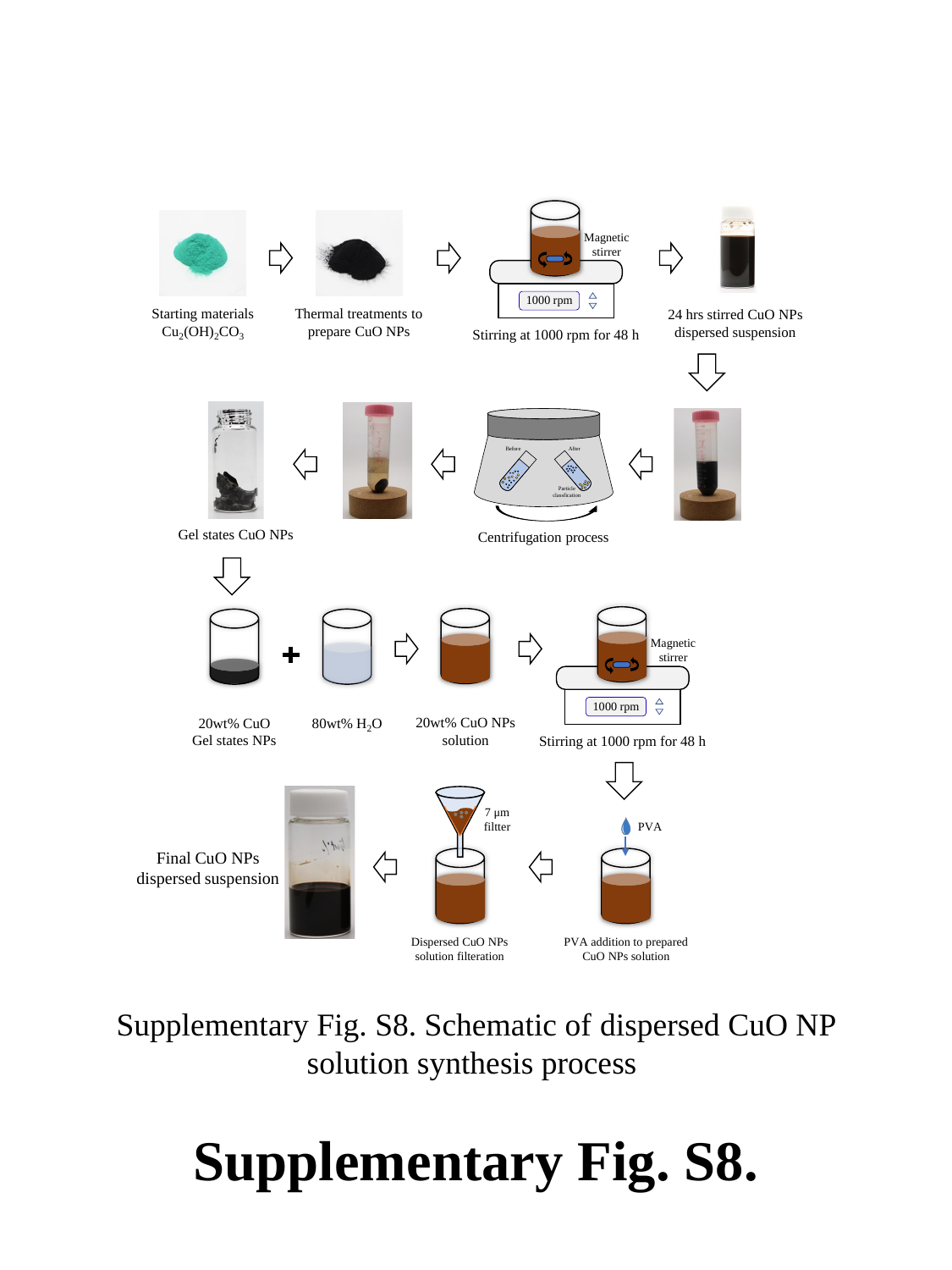

Supplementary Fig. S8. Schematic of dispersed CuO NP solution synthesis process
Supplementary Fig. S8.

## Slide 9
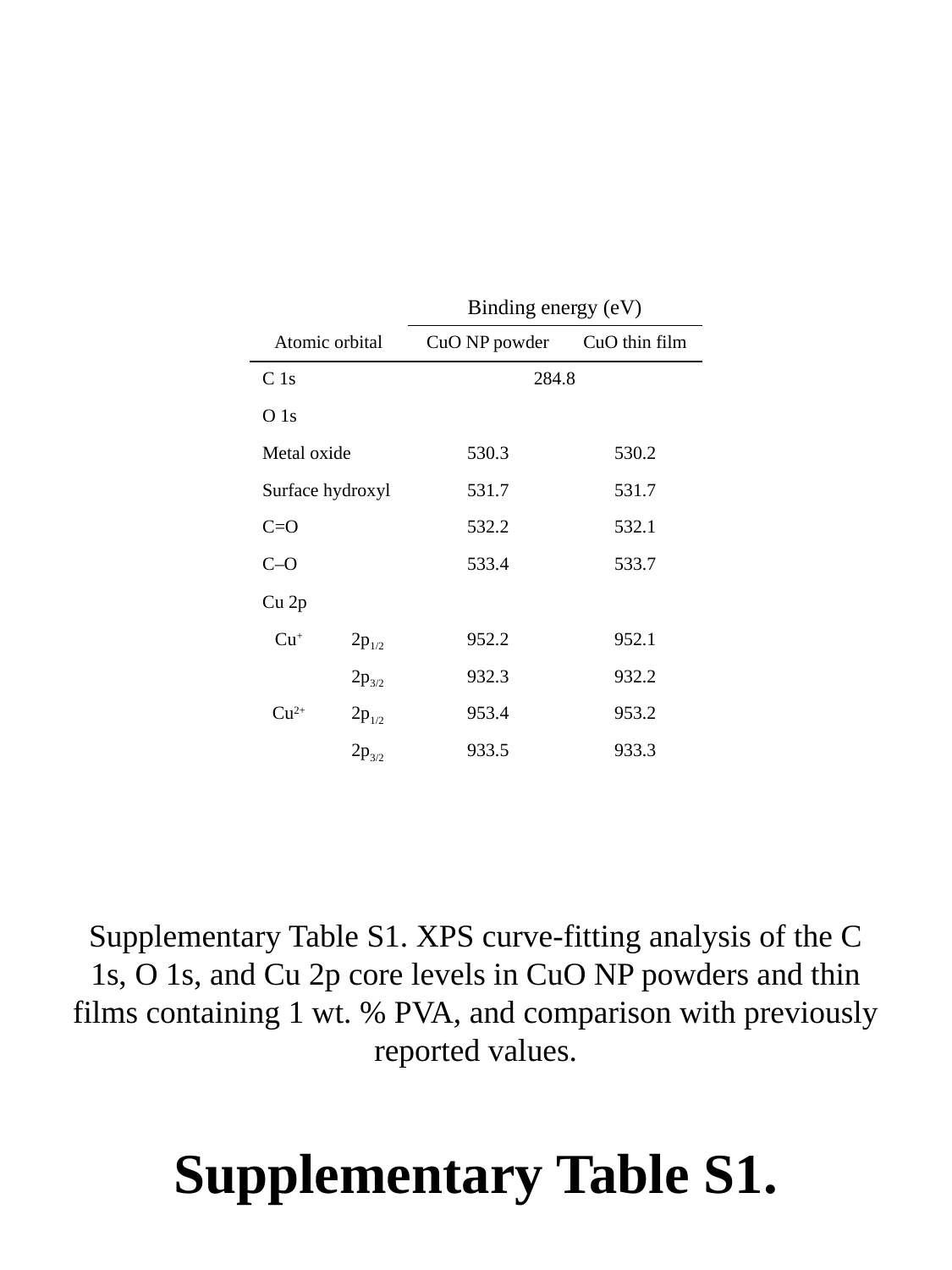

| | | Binding energy (eV) | |
| --- | --- | --- | --- |
| Atomic orbital | | CuO NP powder | CuO thin film |
| C 1s | | 284.8 | 284.8 |
| O 1s | | | |
| Metal oxide | | 530.3 | 530.2 |
| Surface hydroxyl | | 531.7 | 531.7 |
| C=O | | 532.2 | 532.1 |
| C–O | | 533.4 | 533.7 |
| Cu 2p | | | |
| Cu+ | 2p1/2 | 952.2 | 952.1 |
| | 2p3/2 | 932.3 | 932.2 |
| Cu2+ | 2p1/2 | 953.4 | 953.2 |
| | 2p3/2 | 933.5 | 933.3 |
Supplementary Table S1. XPS curve-fitting analysis of the C 1s, O 1s, and Cu 2p core levels in CuO NP powders and thin films containing 1 wt. % PVA, and comparison with previously reported values.
Supplementary Table S1.

## Slide 10
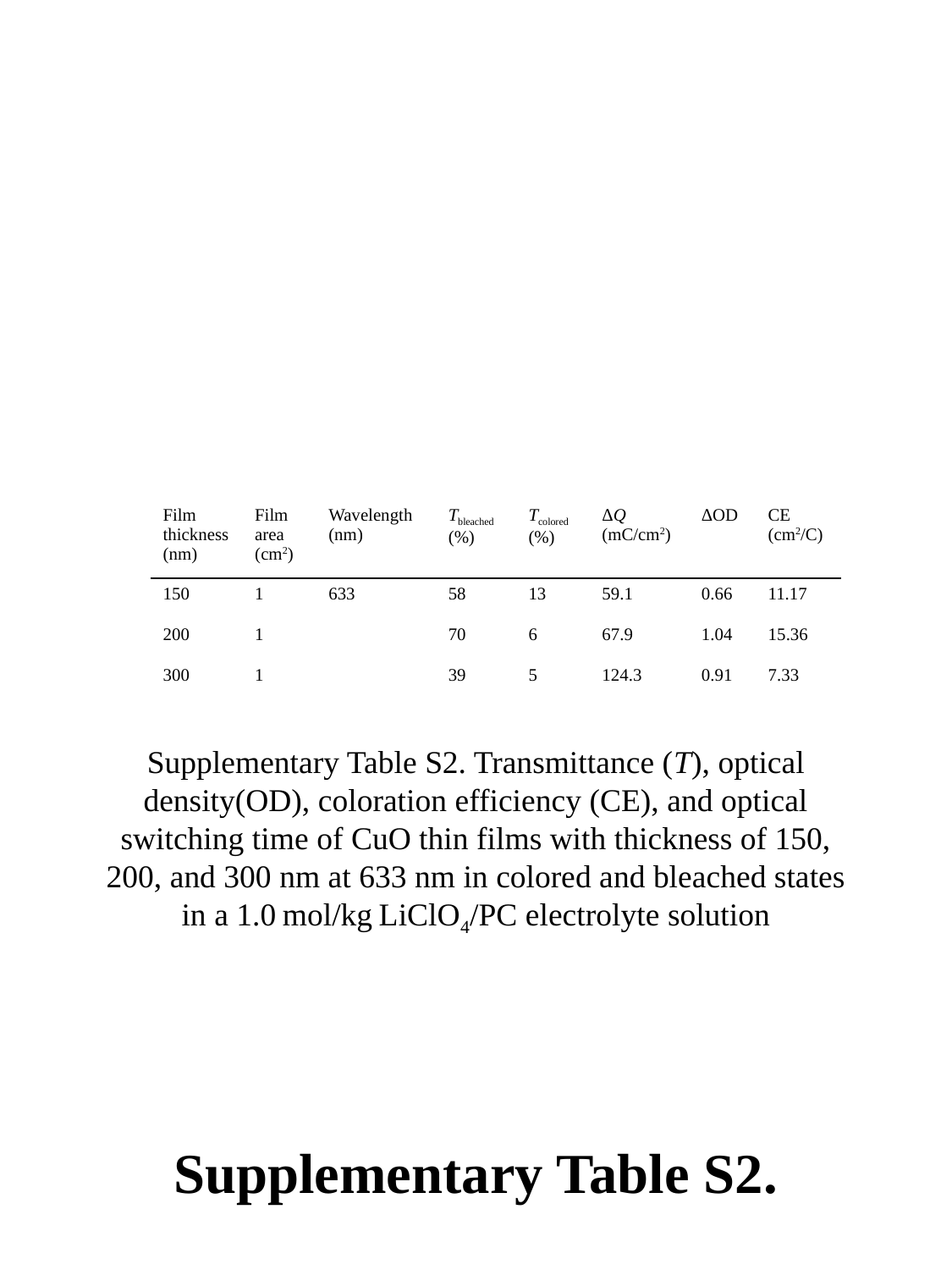

| Film thickness (nm) | Film area (cm2) | Wavelength (nm) | Tbleached (%) | Tcolored (%) | ΔQ (mC/cm2) | ΔOD | CE (cm2/C) |
| --- | --- | --- | --- | --- | --- | --- | --- |
| 150 | 1 | 633 | 58 | 13 | 59.1 | 0.66 | 11.17 |
| 200 | 1 | | 70 | 6 | 67.9 | 1.04 | 15.36 |
| 300 | 1 | | 39 | 5 | 124.3 | 0.91 | 7.33 |
Supplementary Table S2. Transmittance (T), optical density(OD), coloration efficiency (CE), and optical switching time of CuO thin films with thickness of 150, 200, and 300 nm at 633 nm in colored and bleached states in a 1.0 mol/kg LiClO4/PC electrolyte solution
Supplementary Table S2.
